# Supplementary material for: A comparison of two types of contrast media used in endoscopic retrograde cholangiopancreatography: A retrospective study
Source: PLoS One. 2023 Jan 6;18(1):e0280279. doi: 10.1371/journal.pone.0280279 (PMC9821475; doi:10.1371/journal.pone.0280279)
Supplement: Attachment — Submitted filename: PONE20220901_response_20221016.docx [file pone.0280279.s003.docx]

Reviewer #1: This is a retrospective study of contrast medium for ERCP.
1. As the authors admitted, the study results were highly biased due to its retrospective nature. The authors may want to perform multivariable analysis or propensity score matching to reduce biases.

**Response:** Thank you kindly for your comment and for this valuable advice. Further to your suggestion, we undertook to perform propensity score matching in this study. The manuscript has subsequently been revised as there were no longer differences found in terms of the amylase level and the length of hospital stay. Figure 2 and Table 2 have been revised accordingly.

2. A substantial portion of patients had more than one ERCP in the study cohort. The risk of PEP was low once EST was performed. Please consider a subgroup analysis in cases with native papilla.

**Response:** Thank you for your kind comment. After propensity matching study, there were 107 of 162 cases with naïve papilla in the amidotrizoic acid group and 112 of 162 cases with naïve papilla in the iodixanol group. The table below shows the incidence of PEP according to subgroup. Contrast media did not affect the incidence of PEP.

|  | Amidotrizoic acid | Iodixanol | ***P*-value** |
| --- | --- | --- | --- |
| **Naïve papilla (%)** | 16/107 (15.0) | 12/112 (10.7) | 0.420 |
| **Others (%)** | 1/55 (1.82) | 3/50 (6.00) | 0.345 |

3. What is the primary cannulation technique, WGC or contrast injection?

**Response:** Thank you for this important question. Our primary cannulation technique was contrast injection. I have included details concerning this primary cannulation technique in the METHODS section of the manuscript.

4. Guidewire insertion into the pancreatic duct was a risk factor for PEP (PMID: 34963021, 25442080). Please add this factor in the results and discuss in the context of effect of pancreatic stent for PEP.
**Response:** Thank you for your meaningful comment. After propensity matching study, there were 43 cases in the amidotrizoic acid group with guidewire insertion into the pancreatic duct and 47 cases in the iodixanol group. The table below shows the incidence of PEP according to subgroup. Contrast media was not shown to have affected the incidence of PEP.

|  | Amidotrizoic acid | Iodixanol | ***P*-value** |
| --- | --- | --- | --- |
| **With pancreatic stent (%)** | 2/15 (13.3) | 7/24 (29.2%) | 0.254 |
| **Without pancreatic stent (%)** | 8/28 (28.6) | 7/23 (30.4) | 1.00 |

As you have pointed out, guidewire insertion into the pancreatic duct and the placement of a pancreatic stent are related to the onset of PEP. However, these results have not been described in this manuscript because we investigated the effect of differences in contrast media on the onset of PEP, and because we adjusted for risk factors using propensity score matching.

5. The cost is high in iodixanol. Please consider cost analysis.

**Response:** Thank you for your important comment and suggestion. After propensity score matching studies had been undertaken, there was no longer a difference in the length of hospital stay. The average duration of hospital stay was 13.8 days in the iodixanol/IOCM group and 17.5 days in amidotrizoic acid/HOCM group. Iodixanol (Visipaque 100 ml) is 6,000 yen more expensive than amidotrizoic acid (Urografin 100 ml). In Japan, hospitalization costs approximately 8,000 yen per day, but there was no difference in cost because there was no difference in hospital stay (*P* = 0.44).

6. Table 3. Prophylactic pancreatic duct stent placement (%) was repeated.

**Response:** Thank you for pointing this out. The duplicate information has been deleted, as has our original Table 3, with the information contained now added to Table 2.
