# Supplementary material for: A comparison of two types of contrast media used in endoscopic retrograde cholangiopancreatography: A retrospective study
Source: PLoS One. 2023 Jan 6;18(1):e0280279. doi: 10.1371/journal.pone.0280279 (PMC9821475; doi:10.1371/journal.pone.0280279)
Supplement: Attachment — Submitted filename: PONE_response_20221221.docx [file pone.0280279.s004.docx]

Reviewer #1The article is a study about contrast media of ERCP in order to reduce common and severe complication, ERCP pancreatitis (PEP). The authors compared two types of contrast media as a retrospective study. The article is well-written and the authors responded appropriately to the questions from one reviewer. I reviewed the article as the editor and also one reviewer. I have some questions and I think the manuscript needs some revision. I summarized my opinions.

1. The article did not include the details of the criteria for diagnosis of PEP. The information of the reference was shown, but I recommend the authors should show an easy summary of the criteria. Especially, the level of serum amylase is not always necessary for the diagnosis of PEP. For easy understanding of readers, the authors should make clear the difference between PEP and high serum amylase levels in the manuscript. In addition, the authors should compare the other factors of diagnosis of PEP when the authors compared the serum factor which is just one factor of the diagnosis.

**Response:** Thank you for your comment and the valuable advice. Further to your suggestion, we have added details of the diagnostic criteria for PEP to the “Materials and Methods” section. PEP was diagnosed in patients with all three of the following criteria: abdominal pain, hyperamylasemia (≥3 times the upper limit of normal), and prolonged hospital stay (required fasting for eight or more meals), according to the cited literature criteria. In the amidotrizoic acid group, 10.5% of the patients (17 of 162) had abdominal pain, and in the iodixanol group, 10.5% of the patients (17 of 162) had abdominal pain (P = 1.00). Further, 27.1% of the patients (44 of 162) required fasting for eight or more meals in the amidotrizoic acid group, and 17.9% of the patients (29 of 162) required fasting for 8 or more meals in iodixanol group (P = 0.062).

2. 13.8-17.5 days of hospital stays sound long. I guess the hospital stays may include other treatments like cholecystectomy or rehabilitation. If so, I am not sure the comparison of hospital stays between the two groups has meaning. If the authors wanted to compare the complications of ERCP by focusing on two contrast media, they may use other factors, like the period of high serum amylase level.

**Response:** Thank you for your critical comment and suggestion. As noted by the reviewers, the length of hospital stay is strongly influenced by treatment of comorbidities and other diseases. We could not compare the duration of high amylase levels because the timing of blood tests after endoscopy was different in each case. However, we examined the period during which the subjects were able to start eating after ERCP (number of times of fasting after ERCP). The type of contrast media did not affect the duration of fasting (P = 0.499). We have added the result to the “Results.” sections.

6. Review Comments to the Author

Please use the space provided to explain your answers to the questions above. You may also include additional comments for the author, including concerns about dual publication, research ethics, or publication ethics. (Please upload your review as an attachment if it exceeds 20,000 characters) Reviewer #1: 1. The authors provided a table for patient charcateristics in all cohort alone but please add a table for patient characteristics after propensity matching.

**Response:** Thank you for your comment. We have added new tables about patient characteristics after propensity matching (Table 3).
